# Risk factors for new-onset diabetes mellitus after living donor kidney transplantation in Korea - a retrospective single center study

**DOI:** 10.1186/s12882-016-0321-8

**Published:** 2016-07-29

**Authors:** Hoon Yu, Hyosang Kim, Chung Hee Baek, Seung Don Baek, Soomin Jeung, Duck Jong Han, Su-Kil Park

**Affiliations:** 1 Division of a Nephrology, Asan Medical Center, University of Ulsan College of Medicine, Seoul, South Korea; 2 Division of a Nephrology, Gangneung Asan hospital, University of Ulsan College of Medicine, Gangneung, South Korea; 3 Department of Surgery, Asan Medical Center, University of Ulsan College of Medicine, Seoul, South Korea

**Keywords:** Diabetes mellitus, Kidney Transplantation, Magnesium, Tacrolimus

## Abstract

**Background:**

New-onset diabetes mellitus after transplantation (NODAT) is a serious complication following renal transplantation. The aim of this study was to identify the risk factors for the development of NODAT in Korean transplant patients.

**Methods:**

Recipients who underwent living donor kidney transplantation between January 2009 and April 2012 at Asan Medical Center were reviewed. Diagnosis of NODAT was defined according to the American Diabetes Association criteria.

**Results:**

A total of 418 patients were enrolled. NODAT was diagnosed in 85 (20.4 %) patients within 1 year. By multivariate analysis, old age (odds ratio [OR], 1.05; 95 % Confidence interval [CI]: 1.01–1.08), family history of diabetes mellitus (OR, 2.48; 95 % CI: 1.04–5.94), pre-transplant high serum glucose level (OR, 1.04; 95 % CI: 1.01–1.08), and obesity (OR, 3.46; 95 % CI: 1.55–7.73) were independent risk factors for NODAT.

**Conclusion:**

Old age, family history of diabetes, pre-transplant high plasma glucose level, and obesity are independent factors associated with the development of diabetes after renal transplantation. In contrast, serum magnesium levels and the use of tacrolimus are not associated with the development of NODAT.

**Electronic supplementary material:**

The online version of this article (doi:10.1186/s12882-016-0321-8) contains supplementary material, which is available to authorized users.

## Background

New onset diabetes after transplantation (NODAT) is a common and serious complication of renal transplantation and is associated with poor patient and graft survival rates [1, 2]. A number of factors affect the development of NODAT, high body mass index (BMI), calcineurin inhibitors, corticosteroids, old age, family history of diabetes, hypomagnesemia and cytomegalovirus infection are known risk factors for NODAT [3].

However, data on the risk factors for NODAT in Asian transplant populations are lacking. In this present study, we investigated the risk factors for NODAT in a Korean cohort and compared our results to those of previous studies’.

## Methods

### Patients

We retrospectively reviewed patients, who underwent living donor kidney transplantation at Asan Medical Center between January, 2009 and April, 2012. Patients were excluded if they, 1) were younger than 20 years of age, 2) died within 1 year of transplantation, 3) developed graft loss within 1 year of transplantation, and/or 4) were diagnosed with diabetes before transplantation. Patients were classified as having a diagnosis of diabetes within 1 year or not. This study was approved by the Institutional Review Board of Asan Medical center (S2015-1838-0001).

### Clinical data

A triple regimen of calcineurin inhibitors (tacrolimus or cyclosporine) plus mycophenolate mofetil or azathioprine plus glucocorticoids was used for maintenance immunosuppressant therapy. Drug levels of tacrolimus and cyclosporine were monitored based on through level (C0). The dose of glucocorticoids was recorded according to equivalents dose of prednisolone.

A diagnosis of NODAT was defined according to the American Diabetes Association criteria (a fasting glucose level ≥126 mg/dL, glycosylated hemoglobin [Hb A1c] ≥6.5 %, a two-hour value in an oral glucose tolerance test ≥200 mg/dL, or a random plasma glucose concentration ≥200 mg/dL in the presence of symptoms) or a need for anti-diabetic medications. Pre-transplant serum Mg levels were measured a few hours before transplantation. Obesity was defined as a BMI ≥25 kg/m^2^, because all of our patients were Asian. Post-transplant serum Mg levels were measured at 7 days, 1 month and 3 months after transplantation. The estimated glomerular filtration rate (eGFR) was calculated using Chronic Kidney Disease Epidemiology collaboration (CKD-EPI) formulas.

### Statistical analysis

The demographics and laboratory results were compared between NODAT and non-NODAT patients. Comparisons of continuous values were made using the Student’s t-test, and categorical values by Chi-squared test. Logistic regression analysis was performed to investigate the independent risk factors for NODAT. Variables included in multivariate analysis were selected from the univariate analysis. Statistical analyses were performed using the Statistical Package for the Social Sciences (SPSS) software (SPSS Statistics version 20.0, IBM Corporation, Armonk, NY). *P* value <0.05 was considered to indicate a statistically significant difference.

## Results

During the study period, 567 patients underwent living donor renal transplantation at our hospital. From these, 149 patients were excluded from the study for the following reasons: diabetes mellitus before transplantation (*N* = 114), death within 1 year (*N* = 9), loss to follow-up (*N* = 15), and lost graft function within 1 year (*N* = 11). Finally, 418 patients were included in the analysis. Of these, 333 patients were classified into the non-NODAT group and 85 patients were classified into the NODAT group. The rate of NODAT was 20.4 % at 1 year after transplantation. The majority of patients (*N* = 73, 86 %) developed NODAT within 3 months of transplantation. These findings are consistent with those of previous reports [4].

There were no statistical differences in sex, cold ischemic time, rejection history within 1 year, polycystic kidney disease (PCKD), calcineurin inhibitors regimen, corticosteroids dose and serum magnesium levels between the two groups. Older age, high BMI, family history of diabetes, and high plasma glucose level were more often associated with NODAT than non-NODAT patients. The mean weight change at 3 months after transplant was higher in NODAT patients than in non-NODAT patients. The estimated glomerular filtration rate (eGFR) at 3 years post-transplant was significantly lower in NODAT than non-NODAT patients (Table 1).

**Table 1:** Comparison of patients who developed NODAT to those who did not

	Non-NODAT	NODAT	*P* value
Number of patients (%)	333 (79.6)	85 (20.4)	
Age (years)	40.85 ± 10.54	47.82 ± 10.75	<0.001
Sex, male (%)	176 (52.9)	53 (62.4)	0.143
BMI, pre-transplantation (kg/m^2^)	21.9 ± 3.0	24.1 ± 3.2	<0.001
BMI category, pre-transplantation(%)			
≥ 18.5 kg/m^2^	293 (88.0)	84 (98.8)	0.004
≥ 25 kg/m^2^	50 (15.0)	35 (41.2)	<0.001
≥ 30 kg/m^2^	2 (0.6)	4 (4.7)	0.017
≥ 35 kg/m^2^	1 (0.3)	0 (0.0)	0.797
Weight change			
14 days (kg)	2.94 ± 3.54	3.51 ± 3.02	0.179
3 months (kg)	0.67 ± 4.60	3.02 ± 4.87	0.002
Hypertension (%)	301 (90.4)	79 (92.9)	0.534
Prior kidney transplantation (%)	21 (6.3)	4 (4.7)	0.627
Donor Age (years)	41.9 ± 10.9	42.3 ± 10.6	0.805
Donor Sex, male (%)	181 (54.4)	41 (48.2)	0.332
Family history of diabetes mellitus (%)			
Any first degree relative	49 (14.7)	21 (24.7)	0.036
One parent	38 (11.4)	12 (14.1)	0.147
Both parents	2 (0.6)	1 (1.2)	1.000
Sibling	9 (2.7)	8 (9.4)	0.126
Cold ischemic time (minute)	36.0 ± 19.6	40.0 ± 29.5	0.132
Rejection history within 1 year (%)	24 (7.2)	9 (10.6)	0.365
Polycystic kidney disease (%)	13 (3.9)	7 (8.2)	0.148
Immunosuppressive treatment			
Tacrolimus (%)	230 (68.9)	59 (69.4)	1.000
Cyclosporine (%)	104 (31.1)	26 (30.6)	1.000
Mycophenolate mofetil (%)	273 (81.7)	64 (75.3)	0.181
Azathioprine (%)	30 (9.0)	11 (12.9)	0.306
Corticosteroids (%)	315 (94.3)	83 (97.6)	0.273
Tacrolimus level, 1 month (ng/mL)	8.40 ± 3.01	8.45 ± 3.22	0.924
Tacrolimus level, 3 months (ng/mL)	7.45 ± 2.58	7.25 ± 2.63	0.599
Cyclosporine level, 1 month (ng/mL)	216.1 ± 89.6	219.9 ± 91.1	0.854
Cyclosporine level, 3 months (ng/mL)	180.9 ± 79.6	157.5 ± 63.7	0.189
Corticosteroids dose, 1 month (mg)	14.6 ± 3.90	14.9 ± 6.85	0.667
Corticosteroids total dose for 1 month (g)	1.96 ± 0.37	1.95 ± 0.50	0.912
Corticosteroids dose, 3 months (mg)	10.3 ± 3.12	9.8 ± 3.08	0.187
Corticosteroids total dose for 3 month (g)	2.65 ± 0.50	2.63 ± 0.61	0.802
Mg supplementation, pre-transplantation (%)	3 (0.9)	0 (0)	0.611
Serum magnesium level			
Pre-transplantation (mg/dL)	2.17 ± 0.35	2.22 ± 0.34	0.213
7 days (mg/dL)	1.94 ± 0.21	1.97 ± 0.23	0.270
1 month (mg/dL)	1.80 ± 0.23	1.81 ± 0.23	0.897
3 month (mg/dL)	1.82 ± 0.22	1.84 ± 0.23	0.641
Glucose, pre-transplantation (mg/dL)	88.8 ± 10.4	95.7 ± 13.5	<0.001
Total cholesterol (mg/dL)	155.5 ± 35.7	161.9 ± 32.5	0.134
eGFR, 1 year (mL/min/1.73 m^2^)	64.7 ± 19.7	61.2 ± 17.7	0.152
eGFR, 3 years (mL/min/1.73 m^2^)	65.1 ± 21.5	59.4 ± 19.8	0.028

*BMI* body mass index; *Mg* magnesium; *eGFR* estimated glomerular filtration rate

By multivariate analysis (Table 2), the factors associated with NODAT were found to be old age (odds ratio [OR], 1.05; 95 % Confidence interval [CI]: 1.01–1.08), family history of diabetes (OR, 2.48; CI: 1.04–5.94), pre-transplant high plasma glucose level (OR, 1.04; CI: 1.01–1.08), and obesity (OR, 3.46; 95 % CI: 1.55–7.73).

**Table 2:** Multivariate analysis of factors associated with NODAT

Variable	OR	95 % CI	*P* value
Age (years)	1.05	1.01–1.08	0.013
Family history of DM^a^	2.48	1.04–5.94	0.041
Glucose, pre-transplantation (mg/dL)	1.04	1.01–1.08	0.013
Obesity (BMI ≥25 kg/m^2^)	3.46	1.55–7.73	0.002
Weight change, 3 months	1.05	0.97–1.13	0.240
Use of tacrolimus	1.50	0.69–3.26	0.311
Mg, pre-transplantation (mg/dL)	1.80	0.66─4.90	0.249

*BMI* body mass index; *DM* diabetes mellitus; *Mg* magnesium

^a^Any first degree relative

## Discussion

Our present study findings indicated that 20.4 % of our study patients developed NODAT within 1 year of renal transplantation. This result is consistent with the findings from previous reports [5]. While, all of our patients were Korean, the incidence of NODAT was similar to other studies conducted in different populations. Previous studies have shown that pre-transplant glucose levels are independently associated with the development of NODAT [6]. In our present study, high pre-transplant plasma glucose level was an independent risk factor for the development of NODAT. Old age and a family history of diabetes have also been shown to be important factors in the development of NODAT. It is well known that older age and family history of diabetes increase the risk of developing diabetes mellitus in the general population [7]. In our current study cohort, old age and a family history of diabetes appeared to confer a higher risk of diabetes after renal transplantation.

In the general population, low serum magnesium levels are associated with the development of type 2 diabetes, as shown in large cohort studies [8]. One possible explanation is that Mg deficiency may lower the tyrosine kinase activity of insulin receptors, which can in turn lead to post-receptor insulin resistance. In transplant patients, several studies showed an association between hypomagnesemia and diabetes after transplantation [9, 10]. However, some studies found no relationship between hypomagnesemia and NODAT [11, 12]. Augusto et al. recently reported a study focusing on the pre-transplant rather than the post-transplant serum Mg level, and the risk of the development of NODAT, since post-transplant hypomagenesemia can be a result of numerous confounding factors. The authors found that pre-transplant hypomagenesemia is an independent risk factor for NODAT in kidney transplant recipients. In our present study, mean pre-transplant and post-transplant serum Mg levels were not significantly different between non-NODAT and NODAT patients. We gave Mg supplement to the patients who had hypomagenesemia after transplantation, and this resulted in 83 % of patients getting the Mg supplement during the first two weeks after transplantation. This high incidence of Mg supplementation after transplantation, may have affected our results.

Obesity is a well-known risk factor for NODAT in transplant recipients, as well as for diabetes mellitus in the general population [13]. A high prevalence of obesity has been reported in renal transplant recipients. Obesity is related to insulin resistance and consecutive hyperinsulinism. In our current study, the prevalence of obesity was 15 % in the non-NODAT group and 41 % in the NODAT group. By multivariate analysis, patients who were obese prior to the transplantation had a 3.46-fold higher risk of NODAT. In addition, the mean weight change at 3 months after transplant was higher in the NODAT patients than in the non-NODAT patients. Therefore, weight loss should be encouraged in obese transplant recipients.

Calcineurin inhibitors have been associated with the development of NODAT. Many studies have shown that patients treated with tacrolimus have a higher incidence of NODAT compared to those who were treated with cyclosporine [14]. However, in our present study, the use of tacrolimus did not increase the risk of development of NODAT compared to the use of cyclosporine. Since all patients underwent transplantation after January 2009, the concentration of tacrolimus was relatively low because there has been a trend toward maintaining lower tough levels of tacrolimus, following publication of several studies favoring relatively lower levels. This may have contributed to the fact that the use of tacrolimus did not increase the risk of NODAT. In addition, physicians may have reduced tacrolimus levels in patients with high risk of developing diabetes mellitus, knowing the association of tacrolimus and NODAT.

Our study had some limitations. First, it was a retrospective single center study. Second, the glycosylated hemoglobin level was not performed regularly during the follow-up period due to local regulations in non-diabetic patients. Therefore, the prevalence of NODAT may have been underestimated. Finally, as mentioned above, Mg supplementation could not be controlled, because of the retrospective nature of the analysis.

## Conclusion

NODAT is a well-recognized risk factor for poor graft survival in renal transplantation. Identification of possible modifiable risk factors for NODAT in these patients is essential. Old age, family history of diabetes, pre-transplant high plasma glucose level, and obesity are independent risk factors for the development of NODAT. However, serum magnesium levels and the use of tacrolimus (compared with cyclosporine) are not associated with the development of NODAT.

## Abbreviations

BMI, body mass index; CI, confidence interval; CKD-EPI: chronic kidney disease; epidemiology collaboration formulas; DM, diabetes mellitus; eGFR: estimated glomerular; filtration rate; NODAT, new onset diabetes after transplantation; OR: odds ratio; PCKD: polycystic kidney disease

## Additional file

Additional file 1: Supporting information file. Raw data of study population. (XLSX 185 kb)
